# Luminescent Thermometer Based on a Praseodymium(iii) Cyanide-Based Metal–Organic Framework

**DOI:** 10.1021/acs.inorgchem.4c04436

**Published:** 2024-12-19

**Authors:** Nikolia Lalioti, Eleni Zygouri, Vassilis Nastopoulos, Nikos Panagiotou, Carlos D. S. Brites, Luis D. Carlos, Julio Corredoira-Vázquez, Vassilis Tangoulis

**Affiliations:** † Department of Chemistry, University of Patras, Patras 26504, Greece; ‡ Department of Chemistry, University of Cyprus, Nicosia 1678, Cyprus; § Phantom-g, CICECO − Aveiro Institute of Materials, Department of Physics, University of Aveiro, Aveiro 3810-193, Portugal; ∥ Departamento de Química Inorgánica, Facultade de Química, Universidade de Santiago de Compostela, Santiago de Compostela 15782, Spain; ⊥ Institute of Materials (iMATUS), Universidade de Santiago de Compostela, Santiago de Compostela 15782, Spain

## Abstract

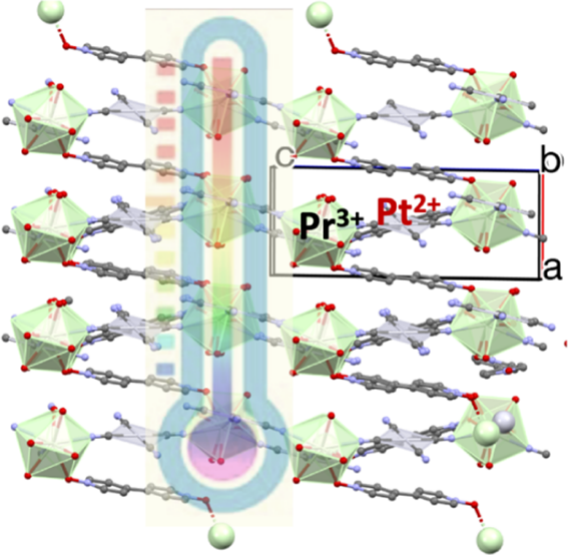

Trivalent lanthanide ions have emerged as promising candidates for precise and remote temperature sensing. Among them, Pr^3+^-based luminescent thermometers remain underexplored, particularly those operating in the near-infrared (NIR) spectral region. This work presents the synthesis and thorough characterization of a novel Pr^3+^-based coordination polymer, {[Pr_2_^III^Pt_3_^II^(CN)_12_(4,4′-bpyO_2_)_4_(H_2_O)_6_]·4H_2_O}_n_ (**1**), as a rare example of Pr^3+^ luminescent thermometry. Coordination between Pr^3+^ ions, cyanido-bridged Pt^2+^ centers, and 4,4′-bpyO_2_ ligands enables efficient energy transfer, producing luminescence in visible and near-infrared regions. The polymer demonstrates distinct temperature-dependent luminescence over a wide range (12–386 K), with relative thermal sensitivities of ≅1%·K^–1^ and a minimum temperature uncertainty of 0.2 K.

## Abstract 2

A novel Pr^3+^-based coordination polymer, {[Pr_2_^III^Pt_3_^II^(CN)_12_(4,4′-bpyO_2_)_4_(H_2_O)_6_]·4H_2_O}_n_, with the linker [Pt(CN)_4_]^2−^ and N-oxide moiety, the organic 4,4′-bipyridine *N*,*N*′-dioxide (4,4′-bpyO_2_) as a promising candidate for luminescent thermometry applications

## Introduction

The trivalent praseodymium ion (Pr^3+^) has emerged as a versatile rare-earth element with exceptional luminescent properties.^1^ Its intricate energy level structure, characterized by a ladder array of energy manifolds, enables the generation of light across a broad spectral range, spanning from the blue and green regions to reddish-orange and near-infrared wavelengths, through downshifting, downconversion, and upconversion processes.^2−5^ Moreover, both *intra-4f* and interconfigurational parity-allowed *5d* → *4f* transitions are discerned, and this luminescence versatility, characterized by both fast and slow decay times, makes Pr^3+^ a promising candidate for applications in lighting, displays, healthcare, security, and sensing.^2,6−8^

A fascinating application is luminescence thermometry, a technique based on the temperature-dependent luminescence properties of phosphors to enable precise and remote detection temperature measurements.^9−11^ Such thermometers hold significant potential for transformative advancements in microelectronics, photonics, biomedicine, and space exploration.^12−17^ Recent trends in luminescence thermometry include exploring materials phase transition to enhance thermometric performance^18−20^ and multimodal temperature determination, which uses the emission intensity of multiple transitions from different Ln^3+^ ions, such as Nd^3+^ and Er^3+^.^21,22^ While Pr^3+^-based luminescent thermometers have been realized in various spectral regions and formats, including nanoscale materials, their application remains less widespread compared to other Ln^3+^ ions (Er^3+^, for instance).^7,23−40^ Notably, all reported Pr^3+^-based thermometers are limited to inorganic materials, where Pr^3+^ serves as a dopant (Table 1).

**Table 1:** Comparison of Maximum Relative Thermal Sensitivity (*S*_m_), the Temperature at which it Occurs (*T*_m_), and the Operational Temperature Range Values of Compound **1** with Those of Selected Inorganic Pr^3+^-based Luminescent Thermometers using Intensity Ratios

Material	S_m_ (% K^–1^)	*T*_m_ (K)	Operational temperature range (K)	ref.
Emission in the UV–visible spectral ranges				
{[Pr_2_^III^Pt_3_^II^(CN)_12_(4,4′-bpyO_2_)_4_(H_2_O)_6_]·4H_2_O}_n_	0.5	275	100–325	This work
YF_3_:Pr^3+^	1.2	293	293–421	(58)
LaF_3_:Pr^3+^	2.3	110	80–320	(26)
KLu_2_F_7_:Pr^3+^	2.8	350	350–600	(68)
β-NaYF_4_:Pr^3+^	4.5	100	50–500	(23)
BaTiO_3_:Pr^3+^	2.3	413	303–413	(69)
Ba_0.9_Ca_0.1_TiO_3_:Pr^3+^	1.6	413	303–413	
Ba_0.7_Ca_0.3_TiO_3_:Pr^3+^	1.4	394	303–413	
Ba_0.6_Ca_0.4_TiO_3_:Pr^3+^	1.4	379	303–413	
Ba_0.4_Ca_0.6_TiO_3_:Pr^3+^	1.3	345	303–413	
Ba_0.3_Ca_0.7_TiO_3_:Pr^3+^	1.1	318	303–413	
Lu_2_SiO_5_:Pr^3+^	3.5	350	275–425	(70)
Lu_2_GeO_5_:Pr^3+^	1.1	515	150–550	
Lu_2_(Ge_0.10_Si_0.10_)O_5_:Pr^3+^	2.2	280	17–600	
Lu_2_(Ge_0.25_Si_0.75_)O_5_:Pr^3+^	1.9	265	17–575	
Lu_2_(Ge_0.50_Si_0.50_)O_5_:Pr^3+^	1.8	120	17–600	
Lu_2_(Ge_0.75_Si_0.25_)O_5_:Pr^3+^	3.5	17	17–600	
Y_3_Al_5_O_12_:0.1%Pr	2.4	250	33–500	(34)
Y_3_(Al_4_Ga_1_)O_12_:0.1%Pr	2.9	261	34–600	
Y_3_(Al_3_Ga_2_)O_12_:0.1%Pr	2.3	345	17–600	
Y_3_(Al_2.5_Ga_2.5_)O_12_:0.1%Pr	3.1	340	30–575	
Y_3_(Al_2_Ga_3_)O_12_:0.1%Pr	2.5	327	75–500	
Y_3_(Al_1.5_Ga_3.5_)O_12_:0.1%Pr	3.6	237	25–450	
Y_3_(Al_1_Ga_4_)O_12_:0.1%Pr	3.6	60	17–275	
YAG:Pr^3+^	0.3	573	293–593	(71)
LaVO_4_:Pr^3+^/Tb^3+^	5.3	300	303–443	(25)
La_2_Ti_3_O_9_:Pr^3+^/Tb^3+^	3.5	300	303–423	
NaLu(MoO_4_)_2_:Pr^3+^/Tb^3+^	2.5	300	303–423	
NaGd(MoO_4_)_2_:Pr^3+^/Tb^3+^	2.1	403	303–486	
NaLu(WO_4_)_2_:Pr^3+^/Tb^3+^	1.5	725	583–783	
La_2_MgTiO_6_:Pr^3+^	1.3	350	77–500	(28)
Na_2_La_2_Ti_3_O_10_:Pr^3+^	2.4	425	296–533	(72, 73)
YNbO_4_:Pr^3+^	1.6	550	300–550	(27)
MgLa_2_TiO_6_:Pr^3+^	1.3	425	300–550	
Sr_2_GeO_4_:Pr^3+^	9.0	22	17–600	(7)
Sr_2_(Ge_0.75_,Si_0.25_)O_4_:Pr^3+^	9.0	65	17–650	(31)
Sr_2_(Ge_0.50_,Si_0.50_)O_4_:Pr^3+^	3.0	225	17–700	
La(Mg_0.402_Nb_0.598_)O_3_:Pr^3+^	0.8	448	298–523	(74)
CaSc_2_O_4_:Pr^3+^	2.5	390	200–490	(75)
LiPr(PO_3_)_4_:Pr^3+^	0.3	164	123–420	(76)
Y_2_Ti_2_O_7_:Pr^3+^	5.3	289	289–573	(33)
Gd_2_Ti_2_O_7_:Pr^3+^	4.6	289	289–573	
LuNbO_4_:Pr^3+^/Tb^3+^	1.3	463	283–493	(77)
CaTiO_3_: 0.6%Pr^3+^+2%NaF	5.2	298	298–523	(78)
CaTiO_3_:Pr^3+^	0.2	50	50–200	(37)
SrSnO_3_/SnO_2_:Pr^3+^	7.7	303	303–473	(79)
Emission in the NIR spectral range				
{[Pr_2_^III^Pt_3_^II^(CN)_12_(4,4′-bpyO_2_)_4_(H_2_O)_6_]·4H_2_O}_*n*_	0.6	290	100–386	This work
LaOF:Pr^3+^	0.8	302	303–593	(38)
LaF_3_:2.20% Pr^3+^,1.45% Yb^3+^	1.1	25	15–105	(32)
NaGdF_4_:0.5% Pr^3+^,20% Yb^3+^	0.3	300	14–330	(39)

Recently, we have embarked on the challenge of developing heterometallic *d–f* MOF-based luminescence thermometers incorporating (a) a red emissive [Co(CN)_6_]^3–^ diamagnetic linker and (b) pyridine derivatives baring N-oxides moieties.^41−43^ We reported a 3D polycyanidometallate luminescence thermometer of the type {Ln_4_Co_4_(CN)_24_(4-benpyo)_17_(H_2_O)]·7H_2_O}_n_ where Ln = Dy^3+^, Eu^3+^ and benpyo = 4-benzyloxy-pyridine N-oxide.^43^ The Co–Eu MOF exhibits luminescence thermometric properties based on the excitation spectra in the 25–325 K range, while the Co–Dy MOF behaves as a luminescence thermometer based on emission features in the 16–350 K range with a maximum relative thermal sensitivity *S*_r_ = 0.6% K^–1^ at 325 K and 2.3% K^–1^ at 240 K respectively. This strategy can be extended to Pr^3+^, as we will showcase next.

In light of these findings, and to develop a Pr^3+^-based luminescent thermometer, we decided to utilize the organic 4,4′-bipyridine *N*,*N*′-dioxide (4,4′-bpyO_2_) as a versatile molecular building block for synthesizing highly dimensional *d–f* coordination systems, including those based on cyanide metal complexes. On the other hand, we have chosen the diamagnetic linker of hexacyanidometallate of Pt^4+^ to sensitize the Pr^3+^ photoluminescence through energy-transfer processes. Until today, a limited number of *d–f* relevant coordination polymers have appeared in the literature, which is mentioned in the following lines. Tanase et al.^44^ detail the formation of *3d–4f* heterometallic coordination polymers using hexacyanometalate building blocks and 4,4′-bpyO_2_ ligands with the formula [{(H_2_O)_5_LPr-NC-M(CN)_5_}(μ-L)]·0.5L·4H_2_O where (*M* = Fe^3+^ or Co^3+^). These complexes feature a novel supramolecular architecture formed through coordinative, hydrogen bonding, and π–π stacking interactions, resulting in infinite zigzag chains. Furthermore, *4f-3d* arrays, forming two-dimensional (2D) corrugated grid-like layers utilizing the 4,4′-bpyO_2_ ligand have been prepared of the type {{Ln^III^(4,4′-bpyO_2_)(H_2_O)_2_M^III^(CN)_6_}2H_2_O}_*n*_ where (Ln = Nd^3+^, Sm^3+^, Gd^3+^, Tb^3+^ and *M* = Fe^3+^, Co^3+^).^45^ The parallel layers in these arrays are interconnected into a three-dimensional (3D) network through a series of hydrogen bonds. The 4,4′-bpyO_2_ ligand is essential for forming and stabilizing the 2D grid-like structure. Trimetallic cyanido-bridged coordination frameworks composed of hybrid coordination layers have also been reported.^46^ These layers are built from cyanido-bridged {Eu^III^[M^II^(CN)_4_]}_n_ square grids and metal–organic {Eu^III^(4,4′-bpyO_2_)}_n_ chains. The frameworks incorporate tetracyanidometallates of Pt^2+^/Pd^2+^ and dicyanidometallates of Au^+^/Ag^+^, resulting in four novel isostructural 2D coordination networks of the type {[Eu^III^(4,4′-bpyO_2_)(H_2_O)_2_][M^II^(CN)_4_]}·[M^I^(CN)_2_]·H_2_O (M^II^ = Pt^2+^, Pd^2+^; M^I^ = Au^+^, Ag^+^). These frameworks feature dinuclear {M^II^M^I^} units formed through metallophilic interactions between the *d*^*8*^ and *d*^*10*^ metal centers. The resulting structures exhibit orange emissive metal-to-metal-to-ligand charge-transfer states, which enhance the red Eu^3+^ photoluminescence at room temperature. The coordination layers are stabilized by both cyanide and 4,4′-bpyO_2_ linkers, with the {M^II^M^I^} units attached through metallophilic interactions.

Recognizing the potential of Pr^3+^-based coordination polymers and metal–organic frameworks for developing NIR optical temperature sensors,^47,48^ and building upon our prior research in lanthanide coordination chemistry, here we investigate the temperature dependence (12–386 K) of a heterometallic praseodymium complex. This study presents the first example of a luminescent thermometer based on a Pr^3+^ cyanide-based metal–organic framework, with emission spanning both the visible and NIR spectral regions.

## Results and Discussion

### Synthesis and IR Spectra

Once we had decided to target Pr^3+^/Pt^4+^/CN^–^/4,4′-bpyO_2_ complexes, we performed several reactions involving various Pr^3+^ starting materials, and different reagent ratios, solvent media (including solvent mixtures) and crystallization methods before arriving at the optimized conditions described in the experimental section. The Pt^4+^ source was chosen to be K_2_[Pt(CN)_6_] to ensure the presence of cyanides in the reaction solution; the N atoms of cyanido ligands in the Pt^4+^ starting material are free to coordinate to Pr^3+^ and we thus expected the isolation of mixed-metal complexes. The hard (HSAB) O atoms of the 4,4′- bpyO_2_ molecule were expected to coordinate to Pr^3+^, thus enforcing the formation of a coordination polymer. Since the Pr^3+^ “salts,” K_2_[Pt(CN)_6_], and 4,4′-bpyO_2_ are all soluble in H_2_O, we focused on the use of this solvent. The 1:1:2 Pr(NO_3_)_3_ 6H_2_O:K_2_[Pt(CN)_6_]:4,4′-bpyO_2_ reaction mixture in warm H_2_O produced amorphous white solid, which was redissolved to give pale green crystals of {[Pr_2_^III^Pt_3_^II^(CN)_12_(4,4′-bpyO_2_)_4_(H_2_O)_6_]·4H_2_O}_*n*_ (**1**) in moderate yield. Subsequent single-crystal X-ray crystallography revealed (*vide infra*) that the oxidation state of platinum has changed from Pt^4+^ to Pt^2+^. This result was unexpected for two reasons: (i) the reaction (which is fully reproducible) took place in the standard aerobic laboratory atmosphere, and (ii) pyridine N-oxides (including 4,4′-bpyO_2_) are oxidants^49^ and can affect a diverse range of powerful oxidative transformations (alkyne, allene and carbene oxidations, among others). Pyridine N-oxides and their derivatives are also reactive compounds, giving a plethora of reactions, e.g., regioselective functionalization of C–H bonds.^49^ Since the ligand 4,4′-bpyO_2_ was found intact in compound **1** and *assuming that this is also the situation in solution*, the only logical explanation is that H_2_O is oxidized by Pt^4+^, giving H^+^ and O_2_ resulting in the reduction of Pt^4+^ to Pt^2+^ (eq 1). Recent results have shown that a high oxidation state of Pt, namely Pt^4+^, is favorable for the oxygen evolution reaction (OER) activity and the formation of high-valent Pt is a key in fabricating an active OER catalyst.^50^ Thus, the reaction can be written in the simplified balanced eq (Eq 2). The italicized H_2_O molecules in the reactants indicate oxidized ones. The presence of the cyanides left in the form of HCN in the products is justified by its weak acidity in aqueous solution (p*K*_a_ = 9.31). Thus, its conjugate base (CN^–^) is much stronger than NO_3_^–^, and the latter ions simply counterbalance the positive charges of potassium cations.
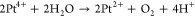
1
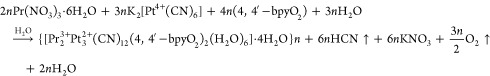
2

In the IR spectrum of compound **1** (Figure S1), the medium-intensity bands at 3557 and 3385 cm^–1^ are assigned to the symmetric O–H stretching vibration, *v*(OH), of the H_2_O molecules (coordinated and lattice).^51^ The δ(OH) vibration appears in the 1635 cm^–1^. The *v*(CH) mode is located at 3115 cm^–1^. The bands in the 1555–1425 cm^–1^ are attributed^52^ to the stretching vibrations of the heterocyclic rings of 4,4′-bpyO_2_.^53^ The bands at 1254 and 1235 cm^–1^ are assigned^52,53^ to the *v*(NO) modes of the 4,4′-bpyO_2_ ligands. The appearance of two bands probably reflects the existence of two types of 4,4′-bpyO_2_ ligands in the complex (*vide infra*). The existence of terminal (Pt—C≡N) and bridging (Pt—C≡N—Pr) cyanido groups in compound **1** are manifested by the bands at 2155 and 2138 cm^–1^, the former being assigned to *v*(C≡N)_bridging_ and the latter to *v*(C≡N)_terminal_.^54^

### Description of Structure

**Figure 1:**
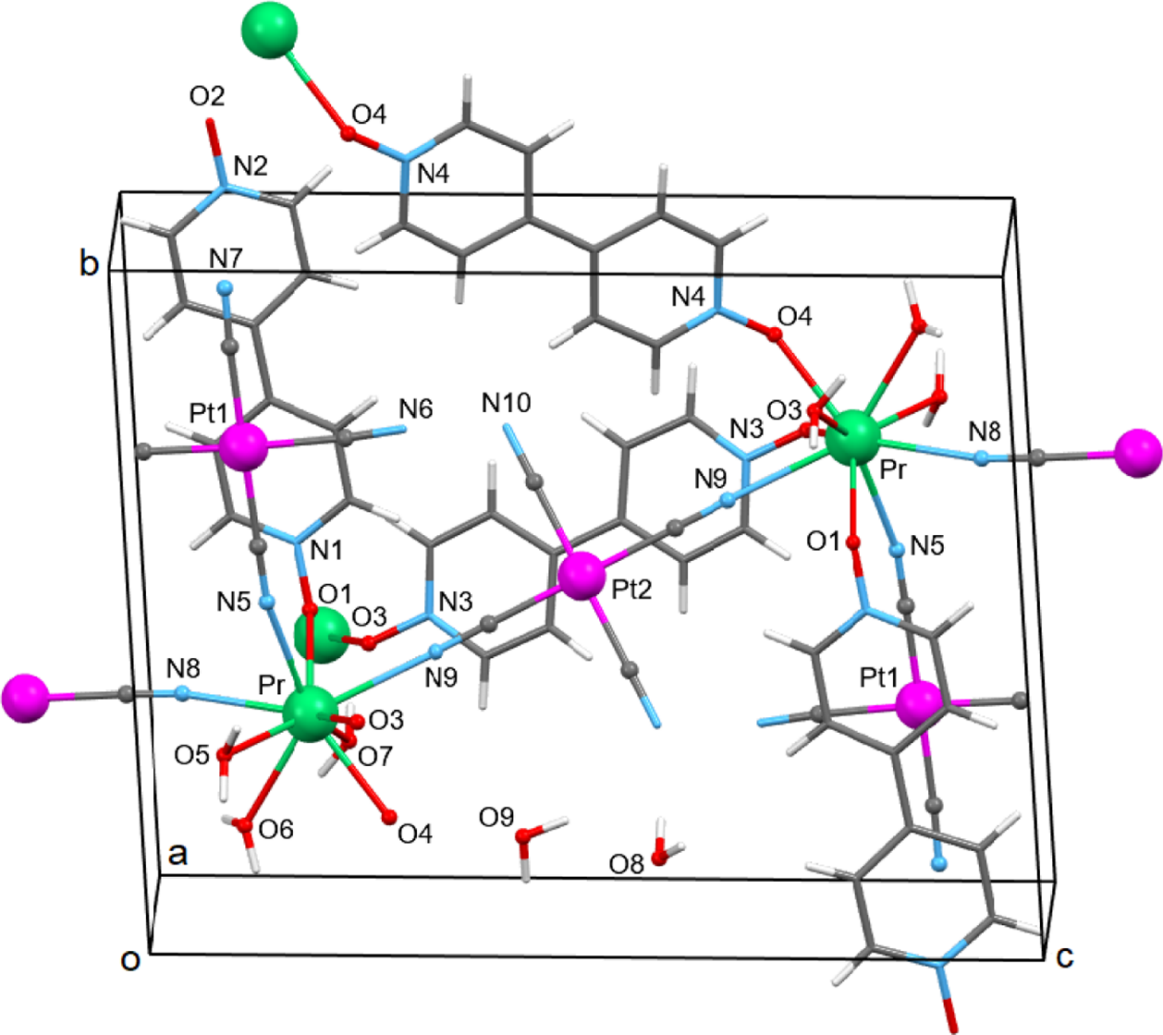
Partially labeled plot of [Pr_2_Pt_3_(CN)_12_(4,4′-bpyO_2_)_4_(H_2_O)_6_]·4H_2_O unit of the polymeric 3D structure of compound **1**. Coordinated atoms have been drawn in ball-and-stick representation. Color code: Pr, green; Pt, magenta; O, red; C, gray; N, blue.

**Figure 2:**
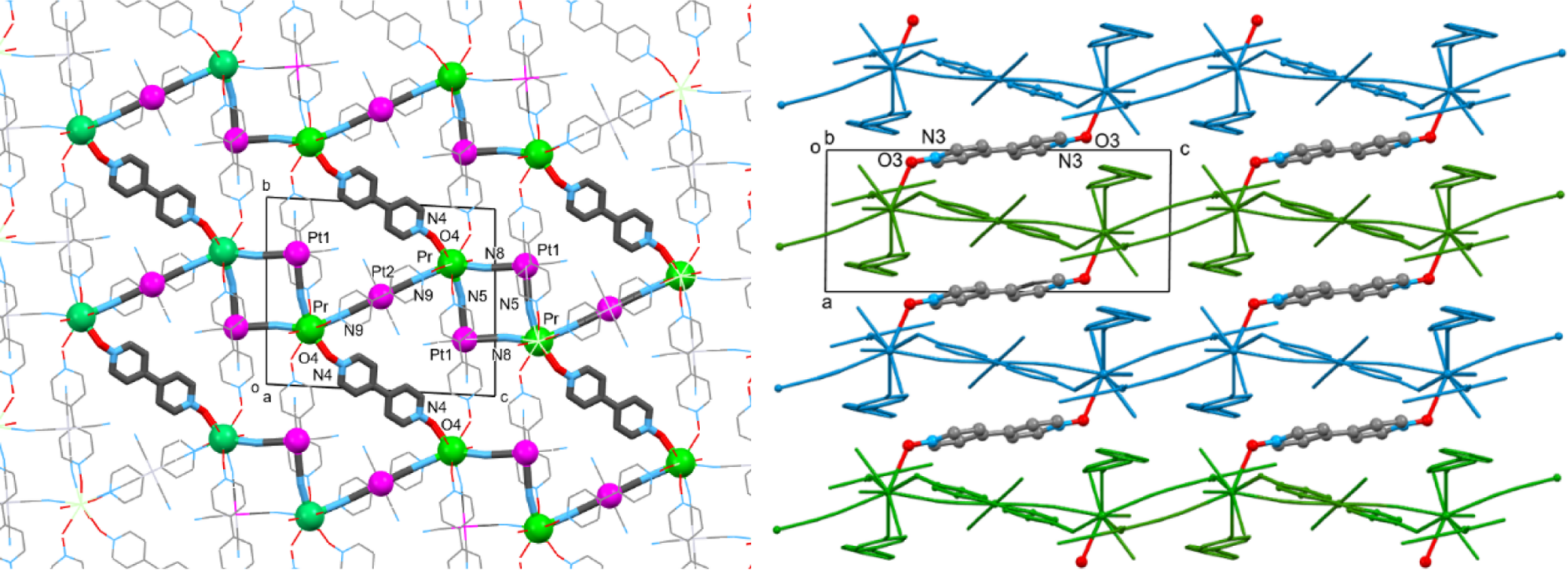
(Left) View of a supramolecular layer of structure of compound **1** parallel to the *bc* plane, containing the 2D network of rings. Each ring includes six Pr^3+^ and four Pt^2+^ atoms—the atoms participating in the rings are highlighted in bold. H atoms and lattice H_2_O molecules are not shown. Color code: Pr, green; Pt, magenta; O, red; C, gray; N, blue. (Right) View of the rings in adjacent parallel planes linked along an axis via another centrosymmetric organic ligand (N3/O3) to a 3D polymeric architecture.

The crystal structure of compound **1** was determined by single-crystal X-ray crystallography. Structural plots are shown in Figures 1 and 2, while numerical data are listed in Tables S1, S2, and S3.

Compound **1** is a 3D coordination polymer crystallizing in the triclinic space group *P*1̅. The asymmetric unit comprises one Pr^3+^ and one and a half Pt^2+^ atoms. Pt1 lies in a general position, and Pt2 lies in an inversion center in the middle of the unit cell.

The organization of the polymer is based on elongated zigzag rings, each consisting of six Pr^3+^ atoms, four [Pt(CN)_4_]^2–^ ions, and two 4,4′-bpyO_2_ ligands. Each [Pt(CN)_4_]^2–^ anion bridges, through two of its cyanido groups, two Pr^3+^ centers forming Pr^III^—N≡C—Pt^II^ moieties. In turn, the centrosymmetric 4,4′-bpyO_2_ ligands, bearing two O4 atoms, also bridge two Pr^3+^ centers of the ring via their O atoms (η^1^*:η*^1^*:μ*_2_) mode (Figure 2, left). The rings share common edges, creating a 2D network parallel to the *bc* plane. The rings in adjacent parallel planes are linked along the *a-axis via* another centrosymmetric organic ligand (N3/O3) to 3D polymeric architecture (Figure 2, right). The 4,4′-bpyO_2_ molecule bearing the oxygen atoms O1 and 2 is only terminally coordinated (η^1^) to the Pr^3+^ atom (through O1) and thus does not participate in the ring formation of the polymeric expansion of the complex. Each Pr^3+^ atom of the [Pr_2_Pt_3_(CN)_12_(4,4′-bpyO_2_)_4_(H_2_O)_6_] unit is coordinated by three O atoms (O1, O3, O4) belonging to three 4,4′-bpyO_2_ molecules, three N atoms (N5, N8, N9) of three η^1^*:η*^1^*:μ*_2_ cyanido groups and three aqua ligands (O5, O6, O7) giving a 9-coordination geometry at each Pr^3+^ atom (Figure 1). Thus, the coordination spheres of metal centers are of the {Pr^III^N_3_O_3_Ο_3_′} and {Pt^II^C_4_} types.

The Pt1···Pt2 (two types), Pt1···Pr and Pt2···Pr distances are 7.091(2) and 11.019(2), 5.734(2) and 5.747(2)Å, respectively. The Pt(1,2)–C bond lengths (∼2.00 Å) are typical of platinum(II)-cyanido bonds.^41^ The Pt—C≡N subunits in the [Pt(CN)_4_]^2–^ ions are practically linear, the corresponding angles being in the 176.2(2)–178.7(2)^o^ range. The coordination geometry at the Pt^2+^ centers is almost perfect square planar, the *trans* C–Pt^II^–C angles being 177.2(2) and 177.9(2)^o^ for Pt1 and strictly 180° (by symmetry) for Pt2. The coordination polyhedron of the 9-coordinate Pr^3+^ atom was evaluated by applying the program SHAPE.^55,56^ According to this program, the {Pr^III^N_3_O_3_Ο_3_′} polyhedron is best described as a spherical capped square antiprism (idealized *C*_4*v*_ symmetry) with the smallest CShM value of 0.627 (Table S3); the next closest value is 0.704 corresponding to a spherical tricapped trigonal prismatic geometry (idealized *D*_3*h*_ symmetry). The same conclusions are also reached by applying the angular criteria established by Kepert.^57^

The crystal structure compound **1** is stabilized by relatively strong H bonds with the coordinated and lattice H_2_O molecules as donors, and some cyanido nitrogen, 4,4′-bpyO_2_ oxygen, and lattice H_2_O molecules as acceptors (Table S4). In addition, several weak C–H···O/N and Pt^2+^···π interactions complement the rigidity of the crystal structure.

Compound **1** is only the third member of a small group of complexes that simultaneously contain Ln^3+^, Pt^2+^, CN^–^ and 4,4-bpyO_2_ metal ions and ligands. The previously described compounds are {[Eu^3+^Pt^2+^(CN)_4_(4,4′-bpyO_2_)(H_2_O)_2_]·[M^I^(CN)_2_]·H_2_O}_*n*_,^46^ were M^I^ = Ag^+^, Au^+^. However, compound **1** is unique because it is 3D and neutral. On the contrary, the Eu^3+^ complexes are cationic 2D coordination polymers and, in addition, they comprise a monovalent metal (Ag^+^, Au^+^) which, in the form [M^I^(CN)_2_]^−^, counterbalances the positive charge; being neutral, compound **1** is lacking a third metal ion.

### Luminescence Studies

**Figure 3:**
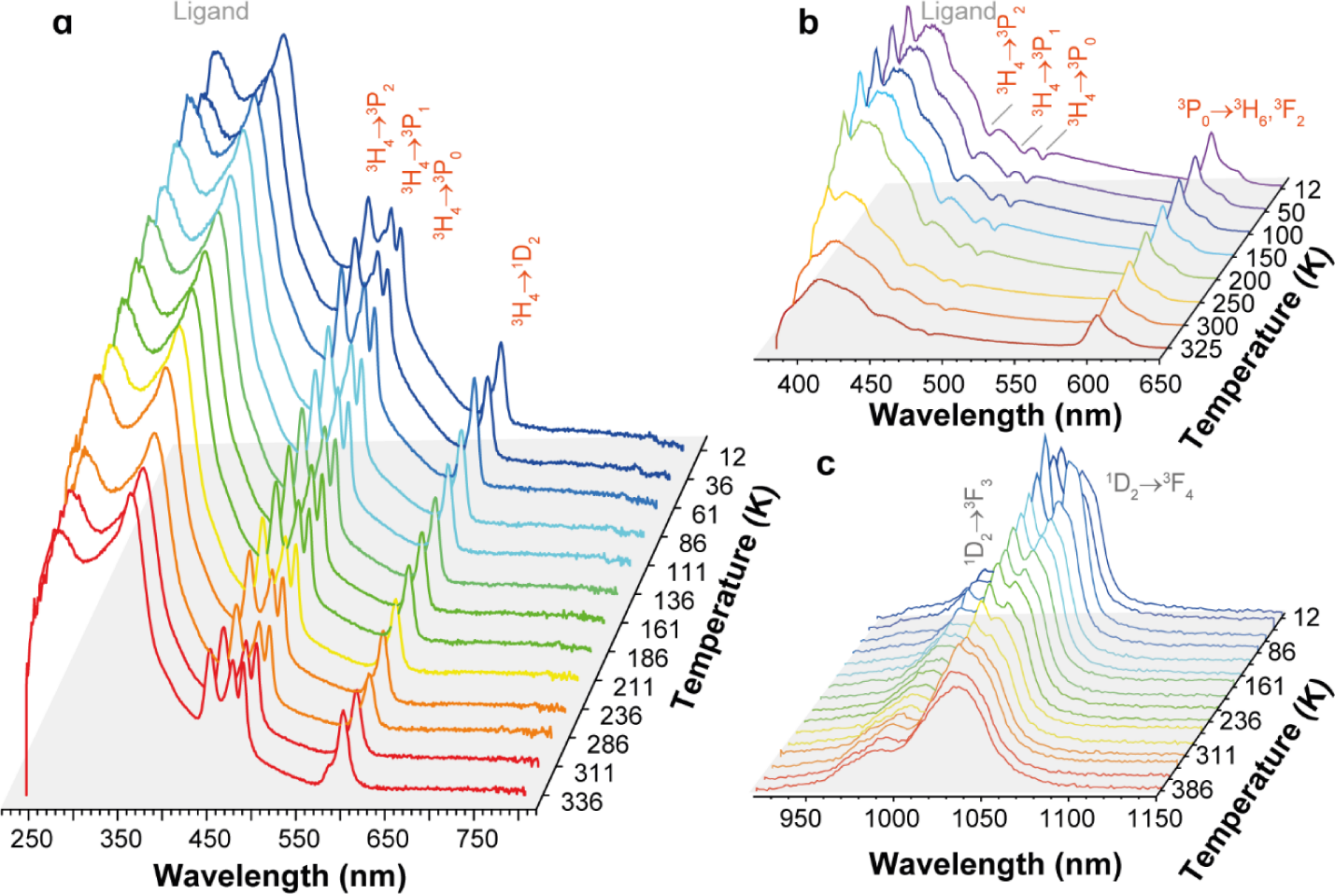
Temperature-dependent photoluminescence of compound **1**: (a) Excitation spectra monitoring the ^1^D_2_ → ^3^F_4_ transition at 1024 nm, (b) Visible emission spectra (excited at 340 nm), and (c) NIR emission spectra (excited at 352 nm).

The excitation spectrum of compound **1** (Figure 3) displays a broad ligand-based band spanning the 250–550 nm region, overlapping with four narrow Pr^3+^ bands ascribed to the ^3^H_4_ → ^3^P_2,1,0_, ^1^D_2_ transitions.^47,58^

**Figure 4:**
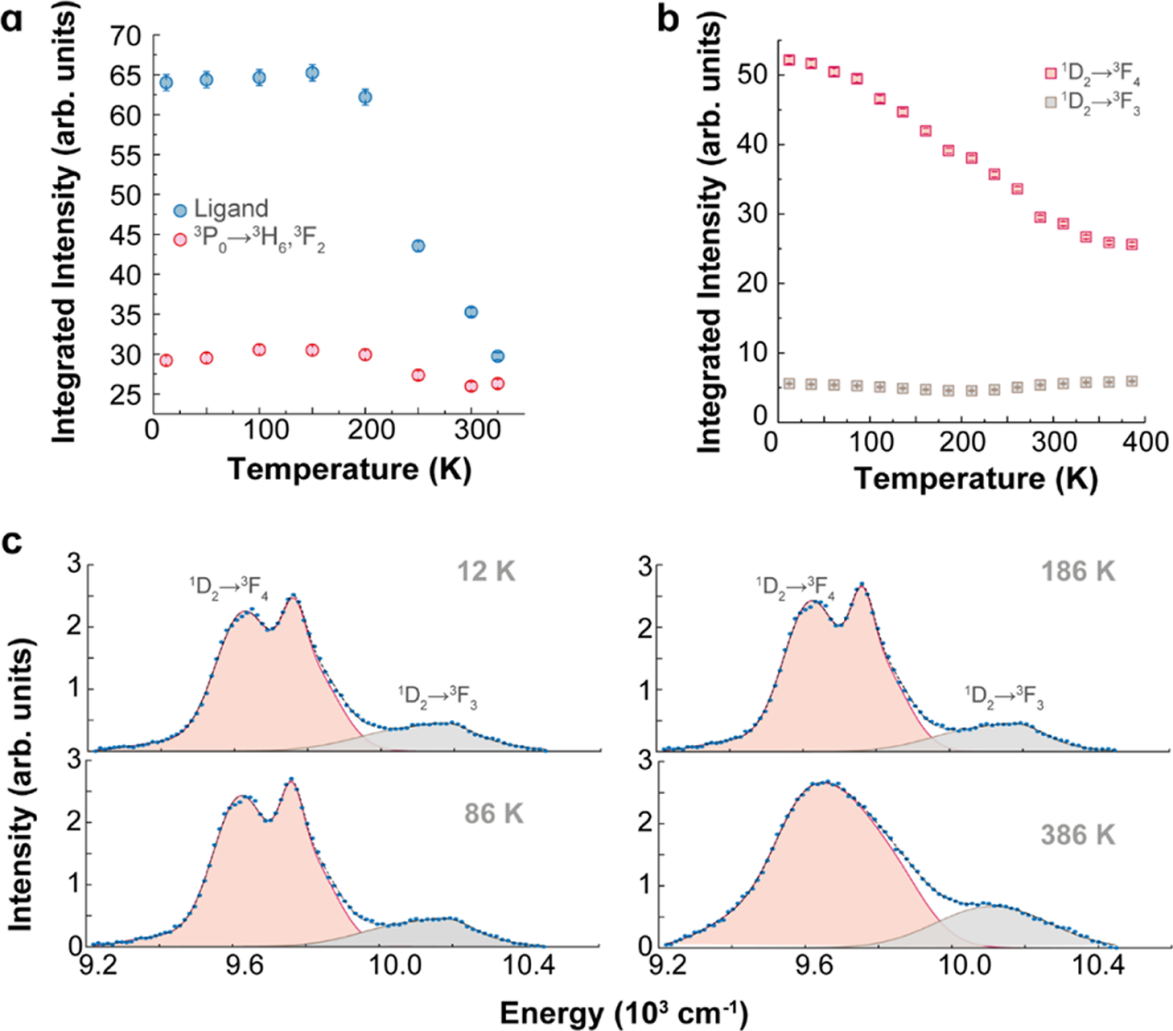
(a) Integrated intensities of the ligand broadband emission and the ^3^P_4_ → ^3^H_6_,^3^F_2_ transitions over the 12–325 K range (calculated from the spectra in Figure 3b). (b) Integrated intensities of the ^1^D_2_ → ^3^F_4_ and ^1^D_2_ → ^3^F_3_ transitions over the 12–386 K range (calculated from the spectra in Figure 3c). (c) Illustration of the deconvolution procedure for selected temperatures, employing four and two Gaussian components to fit the ^1^D_2_ → ^3^F_4_ and ^1^D_2_ → ^3^F_3_ transitions, respectively (details provided in the Experimental Section).

The temperature dependence of the emission spectra of compound **1** in the visible and near-infrared spectral regions is shown in Figure 3b,c, respectively. Across all examined temperatures, the emission spectra exhibited a broad band in the UV–visible spectral region (385–500 nm) attributed to the ligand (Figure S3). In this broadband, Pr^3+^-related self-absorption transitions are observed at about 450, 480, and 490 nm, especially at low temperatures (Figure 3b). This self-absorption phenomenon, known in the literature as the “inner-filter effect”,^59−61^ is observed because the Pr^3+^ ions (the acceptors) have energy levels resonant with the emission of the ligand (the donors), thus absorbing part of the emitted light (an example of a radiative donor-to-acceptor energy transfer). As the temperature increases, the Pr^3+^-related self-absorption transitions and the total integrated intensity of the broadband ligand emission decrease by 50 and 54%, respectively. Additionally, the Pr^3+3^P_0_ → ^3^H_6_,^3^F_2_ transitions, centered at 603 nm, exhibit minimal variation up to 200 K, with only a 10% reduction at higher temperatures (Figure 4a). The distinct temperature dependences of the ligand and Pr^3+^ emissions indicate that thermally activated nonradiative quenching processes predominantly affect the ligands, likely driven by temperature-induced conformational changes. Furthermore, the quenching mechanisms responsible for the temperature dependence of the Pr^3+1^D_2_ → ^3^F_4,3_ transitions in the NIR spectral region (Figure 4b) differ significantly. The ^1^D_2_ → ^3^F_4_ transition intensity decreases by 50% with increasing temperature, whereas the ^1^D_2_ → ^3^F_3_ transition remains temperature-independent. This behavior is likely attributed to the quenching of NIR luminescence dominated by the nearest neighbor CH oscillators.^62^

The integrated areas of the ^1^D_2_ → ^3^F_3,4_ (1025 nm) Pr^3+^ transitions are presented in Figure 4b. To determine the contribution to the total integrated area, we implement a deconvolution procedure illustrated in Figure 4c. We observe a step quenching for the ^1^D_2_ → ^3^F_4_ transition and an almost constant integrated area for the ^1^D_2_ → ^3^F_4_, that acts as a reference for luminescence thermometry.

**Figure 5:**
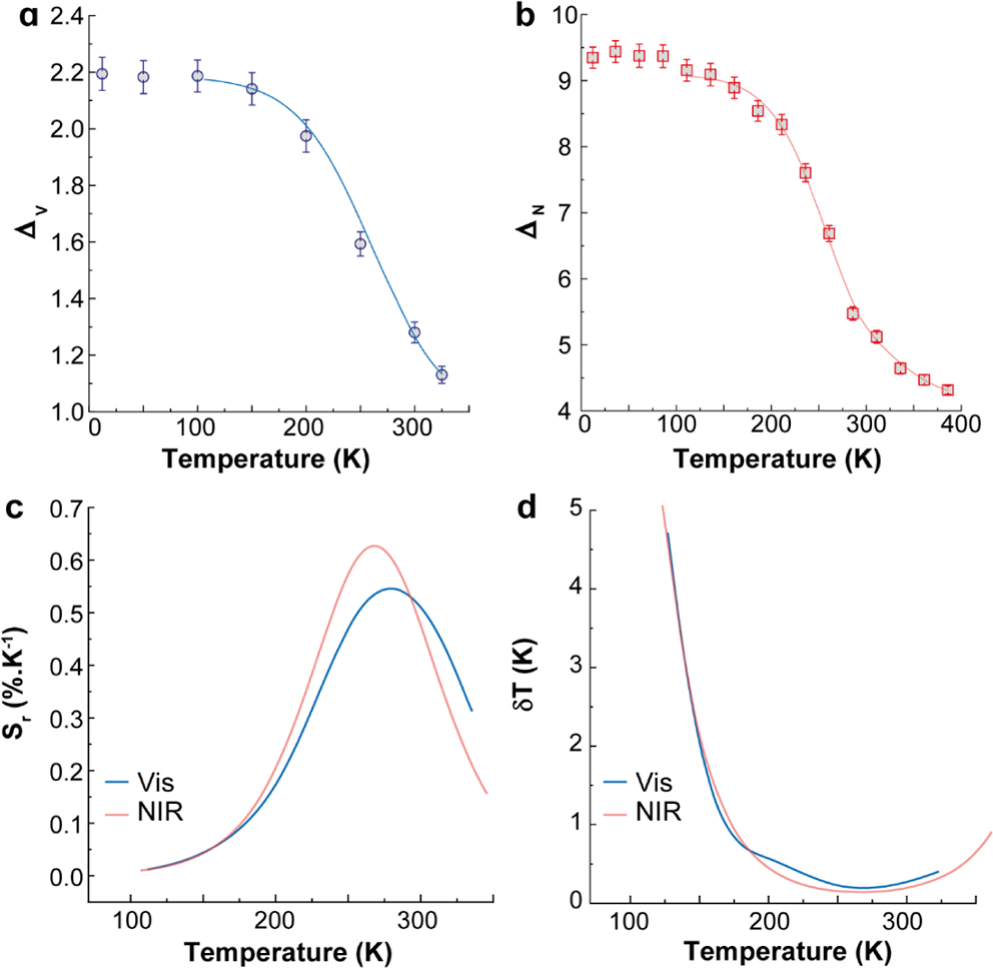
Temperature dependence of the thermometric parameters for compound **1** using the transitions in (a) the visible and (b) the NIR spectral ranges. The lines represent the best fit of eq 3 to the experimental data (*r*^2^ > 0.974). Corresponding temperature dependences of (c) *S*_r_ and (d) *δT*.

To explore compound **1** as a luminescent thermometer, we define ratiometric thermometric parameters using the transitions in the visible (Δ_V_) and NIR (Δ_N_) spectral ranges, defined as Δ_V_ = *I*_1_/*I*_2_ and Δ_N_ = *I*_3_/*I*_4_, where *I*_1_, *I*_2_, *I*_3_, and *I*_4_ correspond to the integrated intensity of the spectra between 380 and 588 nm, 588–650 nm, 900–997.5 nm, and 997.5–1200 nm, respectively. The temperature dependence of *I*_1_, *I*_2_, *I_3_,* and *I*_4_ is presented in Figure 4b,d, while those of Δ_V_ and Δ_N_ are presented in Figure 5a,b. Although ligand-to-metal energy transfer mechanisms often play an important role in describing the temperature-dependence of Ln^3+^ emission intensities,^63−66^ the poor ligand-to-Pr^3+^ energy transfer efficiency observed in this study (Figure 3b) suggests that theoretical calculations will probably not provide significant insights into improving the thermal performance of the luminescent thermometer. Given the limited potential gains of such time-consuming calculations, we instead model the thermal dependence of both thermometric parameter values using a fit to a phenomenological function (eq 3) using OriginLab© software:
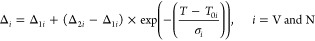
3where Δ_1*i*_ and Δ_2*i*_ are fitting parameters corresponding to the values in the low and high temperatures limit, respectively, *T*_0*i*_ denotes the temperature of the inflection point and *σ*_*i*_ is a parameter controlling the decay rate. The values of the fitting parameters for the best fit of eq 3 to the experimental Δ_V_ and Δ_N_ values are presented in Table S5.

For temperatures below 100 K, the variations in the Δ_V_ and Δ_N_ values are of the same order as their uncertainties, so the luminescent thermometers are out of their operating range.

The thermometric performance of compound **1** was assessed through the typical figures of merit for luminescent thermometers, the relative thermal sensitivity (*S*_r_), and the temperature uncertainty (*δT*)^9,67^ (Figure 5c,d). The maximum *S*_r_ values for compound **1** are 0.63 and 0.55% K^–1^ for visible and NIR spectral ranges, 275 and 290 K, respectively. The corresponding minimum temperature uncertainty values are 0.2 K at the same temperature. Note that the best performance parameters of the thermometer occur near room temperature. The thermometric performance of compound **1** is comparable to those of the previously reported inorganic Pr^3+^-based luminescent thermometers, particularly in the NIR range (Table 1).

## Conclusions

This work presents the synthesis and comprehensive characterization of a novel Pr^3+^-based coordination polymer, {[Pr_2_^III^Pt_3_^II^(CN)_12_(4,4′-bpyO_2_)_4_(H_2_O)_6_]·4H_2_O}_n_, as a promising candidate for luminescent thermometry applications. The reduction of Pt^4+^ to Pt^2+^ under aerobic conditions, facilitated by the redox activity of water molecules, represents a significant finding, providing deeper insights into the redox behavior within this coordination system. The coordination of Pr^3+^ ions with cyanido-bridged Pt^2+^ centers and 4,4′-bpyO_2_ ligands was shown to enable effective energy transfer, resulting in strong luminescence emissions in both the visible and near-infrared (NIR) regions. These luminescent properties underscore the potential of this material for optoelectronic applications.

The application of this polymer as a luminescent thermometric revealed a distinct temperature dependence of the luminescence over a wide temperature range (12–386 K), demonstrating the suitability of this polymer for accurate luminescence-based temperature sensing using emission both in the visible and in the NIR spectral ranges. The relative thermal sensitivity values are slightly below 1%·K^–1^ and the minimum temperature uncertainty is about 0.2 K, showcasing the potential of the material as a promising candidate for applications in fields such as microelectronics, biomedical monitoring, and aerospace applications. Future work will aim to optimize the thermometric performance of this coordination polymer by enhancing its luminescence sensitivity and exploring additional heterometallic configurations to further improve its functional properties. These efforts could expand the utility of Pr^3+^-based luminescent thermometers in diverse technological applications.

## Experimental Section

### Materials, Physical Techniques, and Spectroscopic Methods

All manipulations were performed under aerobic conditions using materials Pr(NO_3_)_3_6H_2_O (Sigma-Aldrich, CAS 15878-77-0) and K_2_[Pt(CN)_6_)]*x*H_2_O (Sigma-Aldrich, CAS 38245–27–1) and 4,4′-bipyridine (98%, Alfa Aesar, CAS 553-26–4) as received. The synthesis of (4,4′-bpyO_2_) was described elsewhere.^80^ H_2_O was distilled in-house. Elemental Analyses were performed by University of Patras instrumental service. FT-IR spectra (4000–450 cm^–1^) were recorded using a PerKin–Elmer 16PC spectrometer with samples prepared as KBr pellets.

### Preparation of {[Pr_2_Pt_3_(CN)_12_(4,4′-bpyO_2_)_4_(H_2_O)_6_]·4H_2_O}_*n*_ (1)

Solid Pr(NO_3_)_3_·6H_2_O (0.028g, 0.065 mmol) was added to a stirred pale yellow warm aqueous solution (0.7 mL) of 4,4′-bpyO_2_ (0.025 g, 0.13 mmol). The solid was soon dissolved and to the resulting solution was added an aqueous solution (0.7 mL) of 0.028 g of K_2_[Pt(CN)_6_] *x*H_2_O; the resulting slurry was stirred at ∼50 °C until dissolution. Storage of the solution in a closed vial at room temperature resulted in the precipitation of a small quantity of an amorphous solid which was soon dissolved. Tiny, pale green needle-like crystals of the product appeared within a period of 1 d. The crystals were collected by filtration, washed with ice-cooled H_2_O (0.3 mL) and dried *in vacuo* over anhydrous CaCl_2_. The yield was ∼40% (based on the Pr^3+^ available). p-XRD diffraction pattern of the product is given in Figure S2. Anal. Calcd for C_52_H_52_N_20_O_18_Pr_2_Pt_3_: C, 29.57; H,2.49; N, 13.27%. Found: C, 29.87; H, 2.44; N, 13.02%. IR (KBr, ṽ/cm^–1^): 3557m, 3385mb, 3115w, 2155w, 2138s, 1635m, 1478s, 1470sh, 1427m, 1320w, 1254m, 1225s, 1178m, 1032m, 872sh, 842s, 700m, 592w, 558s, 524w, 501w, 417m. *Caution!* Hydrocyanic acid (HCN) may be produced by the reactions and all operations should be performed in a closed strong fume cupboard.

### Single-Crystal X-Ray Crystallography

A suitable single-crystal of compound **1**, covered with paratone_–_N oil, was scooped up in a cryo-loop at the end of a copper pin and transferred to a goniostat, where it was cooled under a flow of nitrogen gas at 105(2) K. Diffraction data were collected (*w*-scans) on a RigaKu SuperNova diffractometer using Cu K*a* radiation (λ = 1.5418 Å). Data were collected and processed by the Crysalis CCD and RED software,^81^ respectively. The reflection intensities were corrected for absorption by the multiscan method. The structure was solved using direct methods with SIR92^82^ and refined by full-matrix least-squares on *F*^2^ with SHELXL-2019/3.^83^ Anisotropic refinement was applied for all non-H atoms. Carbon–bound H atoms were included in the model at calculated positions and allowed to ride on their respective carrier atoms (riding model). All H atoms of the coordinated and lattice H_2_O molecules (O5 to O9) were located in difference. Fourier maps and refined isotropically applying soft distance restraints (DFIX). Geometric/crystallographic calculations were carried out using the WINGX,^84^ PLATON,^85^ and OLEX2.^86^ Molecular/packing graphics were prepared with MERCURY.^87^ Some crystallographic data collection and refinement parameters are listed in Table S1.

### Photoluminescence Measurements

The temperature-dependent emission and excitation spectra in the solid state for compound **1** were recorded using a modular double grating excitation spectrofluorometer featuring a TRIAX 320 emission monochromator (Fluorolog-3, Horiba Scientific) coupled with a R298 Hamamatsu photomultiplier and a H9170 Hamamatsu photomultiplier, employing a front face acquisition mode. The excitation source utilized was a 450 W Xe arc lamp. The emission spectra were corrected for detection and optical spectral response of the spectrofluorometer, and the excitation spectra were corrected for the spectral distribution of the lamp intensity using a photodiode reference detector. The temperature was adjusted by a He-closed cycle cryostat coupled to a vacuum system (4 × 10^–4^ Pa), and an autotuning temperature controller (Lakeshore 330, Lakeshore) equipped with a resistance heater. The temperature was measured by a silicon diode cryogenic sensor (DT-470-SD, Lakeshore) with an accuracy of ±0.5 K (12–30 K), ± 0.25 K (30–60 K), and ±0.15 K (60–385 K). All the emission and excitation spectra were recorded only after the temperature indicated in the temperature controller stabilized, ensuring both sample thermalization and a constant temperature throughout the measurement.

### Integrated Areas Determination

A MATLAB code was developed to process and analyze the emission spectra recorded at distinct temperatures. First, the baseline is removed and then the measured emission intensity is converted to energy units (*E*) using the Jacobian transformation, according to the established procedures.^88,89^ The resulting intensity is then integrated numerically over defined energy ranges for the (Pr^3+^) ions and the ligand, calculating the areas under the curves recorded in the UV–vis spectral range. For the emission spectra in the NIR spectral range, we observe a significant overlap between the ^1^D_2_ → ^3^F_4_ and ^1^D_2_ → ^3^F_5_ transitions, and thus a deconvolution procedure was implemented in the same software. The emission spectra are well deconvoluted using six Gaussian functions, in which the 4-lowest energy ones are ascribed to the ^1^D_2_ → ^3^F_4_ transition and the remaining 2 to the ^1^D_2_ → ^3^F_3_ one. The corresponding integrated areas result from the area of the fitted curves. Irrespective of the area determination procedure, the uncertainty in determining the integrated area, *I*_*i*_ is estimated using the baseline fluctuations. It is calculated as *δI*_*i*_ = *I*_*i*_ × 1/SNR, where SNR (signal-to-noise ratio) is determined as the ratio between the maximum intensity of the spectrum and the noise fluctuations in a spectral region outside the transitions range.^67^

### Thermometric Performance Assessment

The relative thermal sensitivity (*S*_*r*_) of compound **1** was estimated by
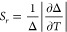
4where Δ is the thermometric parameter and *T* is the temperature. The temperature uncertainty (*δT*), is determined by^12^
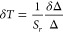
5where *δΔ/Δ* is the relative uncertainty in Δ estimated through:
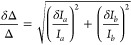
6where *δI*_*i*_/*I*_*i*_ (*i* = *a, b*; *a* = 1 or 4, and *b* = 2 or 3) is calculated by dividing the readout fluctuations of the baseline by the maximum value of each intensity, i.e., *I*_1_ and *I*_2_. As the integrated areas are calculated from different emission spectra,*δI*_1_ = *δI*_2_= and *δI*_3_ = *δI*_4_.

## Supporting Information Available

IR spectrum and p-XRD diffraction pattern. Single crystal diffraction data regarding structure refinement, selected bond lengths/angles and hydrogen-bonding interactions. A table regarding coordination geometry analysis of compound **1** (PDF)
