# Supplementary material for: Luminescent Thermometer Based on a Praseodymium(iii) Cyanide-Based Metal–Organic Framework
Source: Inorg Chem. 2024 Dec 19;64(1):192–201. doi: 10.1021/acs.inorgchem.4c04436 (PMC11734120; doi:10.1021/acs.inorgchem.4c04436)
Supplement: Supplementary file 1 — ic4c04436_si_001.pdf [file ic4c04436_si_001.pdf]

SUPPORTING INFORMATION

Luminescent Thermometer based on a Praseodymium (III) cyanide-based metal-organic framework

Nikolia Lalioti,[a] Eleni Zygouri,[a] Vassilis Nastopoulos,[a] Nikos Panagiotou,[b] Carlos D. S. Brites,[c] Luis D. Carlos,[c*] Julio Corredoira-Vázquez,[c] Vassilis Tangoulis [a*]*

[a] Department of Chemistry, University of Patras, 26504, Patras, Greece.

[b] Department of Chemistry, University of Cyprus, Nicosia, Cyprus

[c] Phantom-g, CICECO – Aveiro Institute of Materials, Department of Physics, University of Aveiro, 3810-193 – Aveiro, Portugal.

[d] Departamento de Química Inorgánica, Facultade de Química, Universidade de Santiago de Compostela, 15782 Santiago de Compostela, Spain.

[e] Institute of Materials (iMATUS), Universidade de Santiago de Compostela, 15782 Santiago de Compostela, Spain.

IR Spectroscopy

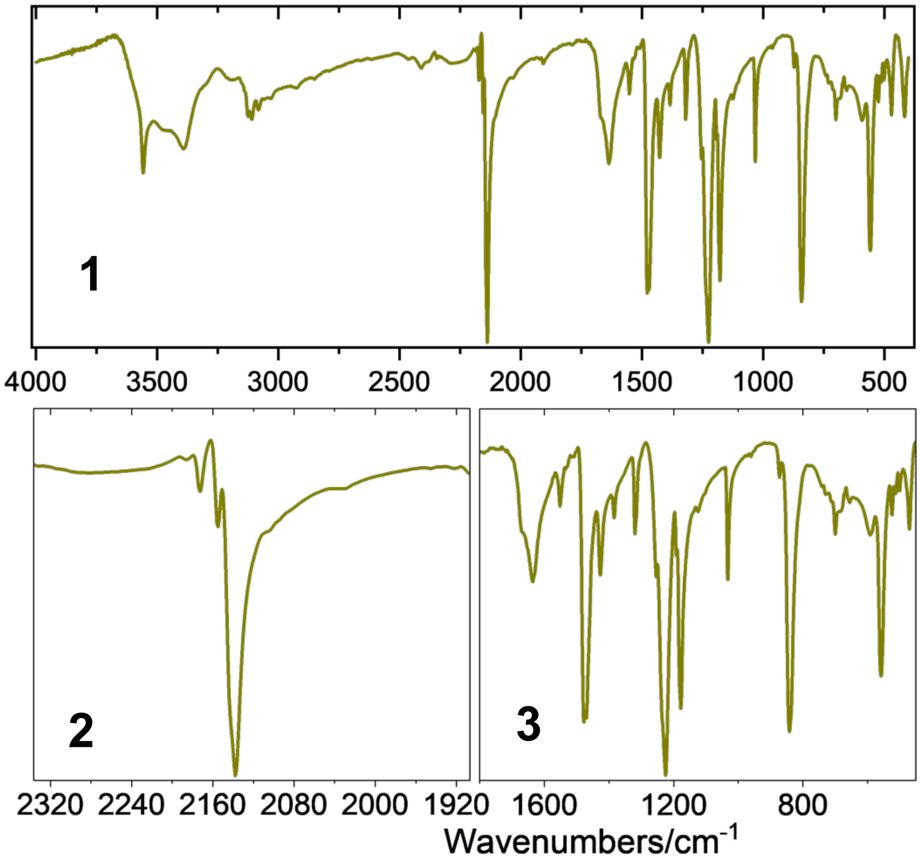

Figure **S1.** IR spectrum of compound **1** between **(1)** 4000 – 450 cm^{-1}; **(2)** 2350 – 1900 cm^{-1}; and **(3)** 1700 – 480 cm^{-1}.

p-XRD Diffraction

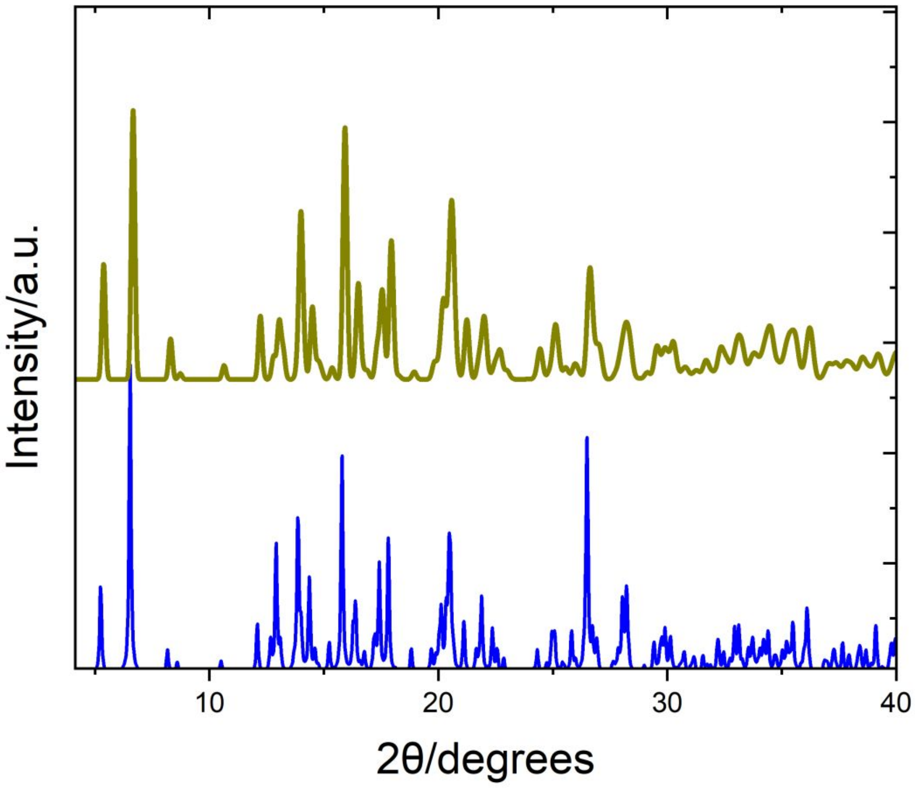

Figure **S2.** pXRD diffraction pattern of compound **1 (** olive color) and the calculated one based on the single crystal diffraction study of compound **1.**

Single Crystal Diffraction

Table S1. Crystal data and structure refinement for compound **1**.

Chemical formula	$C_{52}H_{44}N_{20}O_{14}Pr_2Pt_3 \cdot 4(H_2O)$
Formula weight	2112.22
Temperature [K]	105(2)
Crystal system	triclinic
Space group	*P*-1
a [Å]	6.9083(4)
b [Å]	13.6465(7)
c [Å]	16.8454(6)
α [deg]	92.922(3)
β [deg]]	90.244(4)
γ [deg]	97.453<br>(4)
Volume [Å^3]	1572.53(13)
Z	1
ρ_{calc} [g cm^{-3}]	2.230
F(000)	1000
Radiation type, μ [mm^{-1}]	Cu*Kα*, 24.56
Collected reflections	9505
Unique reflections	5598
R_{int}	0.029
θ [deg]/completeness (%)	67.0/99.9
Data/parameters/restraints	5598/460/30
Final R_1, $wR_2(F^2)$ [$I > 2\sigma(I)$]	0.028, 0.070
Final R_1, $wR_2(F^2)$ (all data)	0.029, 0.071
Goodness-of-fit on F^2	1.03
$\Delta\rho_{max}$, $\Delta\rho_{min}$ (e Å^{-3})	1.66, −2.11
CCDC number	**2386784**

Table S2. Selected bond lengths [Å] and angles [°] for compound **1**.

Pt1—C21	1.998 (5)	C24^{i}—Pt1—C23	90.72 (17)
Pt1—C22	2.008 (5)	C24^{i}—Pt1—C21	89.08 (17)
Pt1—C23	1.998 (5)	C23—Pt1—C21	177.91 (17)
Pt1—C24^{i}	1.992 (5)	C24^{i}—Pt1—C22	177.16 (17)
Pt2—C26	1.991 (5)	C23—Pt1—C22	87.36 (17)
Pt2—C25	1.997 (5)	C21—Pt1—C22	92.76 (17)
Pr—O1	2.490 (3)	C25^{ii}—Pt2—C25	180.0
Pr—O3	2.532 (3)	C26—Pt2—C25	89.30 (18)
Pr—O4	2.446 (3)	C26^{ii}—Pt2—C26	180.0
Pr—O5	2.584 (3)	C26—Pt2—C25^{ii}	90.70 (18)
Pr—O6	2.562 (3)		
Pr—O7	2.473 (3)		
Pr—N5	2.637 (4)		
Pr—N8	2.633 (4)		
Pr—N9	2.626 (4)		
O1—N1	1.316 (5)		
O2—N2	1.332 (5)		
O3—N3	1.332 (5)		
O4—N4	1.320 (5)		

Symmetry codes: (i) −x+1, −y+1, −z+2; (ii) −*x*+1, −*y*+1, −*z*+1.

Table S3. Coordination geometry analysis of compound **1** by the SHAPE v2.1 software.

9-coordinated Praseodymium metal center

EP-9 D_{9h} Enneagon
OPY-9 C_{8v} Octagonal pyramid
HBPY-9 D_{7h} Heptagonal bipyramid
JTC-9 C_{3v} Johnson triangular cupola J3
JCCU-9 C_{4v} Capped cube J8
CCU-9 C_{4v} Spherical-relaxed capped cube
JCSAPR-9 C_{4v} Capped square antiprism J10
CSAPR-9 C_{4v} Spherical capped square antiprism
JTCTPR-9 D_{3h} Tricapped trigonal prism J51
TCTPR-9 D_{3h} Spherical tricapped trigonal prism
JTDIC-9 C_{3v} Tridiminished icosahedron J63

EP-9	OPY-9	HBPY-9	JTC-9	JCCU-9	CCU-9	JCSAPR-9	**CSAPR-9**	JTCTPR-9	**TCTPR-9**	JTDIC-9
36.147	22.314	19.462	15.880	10.421	8.713	2.063	**0.627**	2.508	**0.704**	12.460

TABLE S4. Geometry (Å, °) of the weak hydrogen-bonding interactions in compound **1.**

D–H···*A*	*D*–H	H···*A*	*D*···*A*	*D*–H···*A*
O5—H5A···O2^{i}	0.85 (2)	1.86 (2)	2.691 (4)	166 (5)
O6—H6A···N7^{ii}	0.86 (2)	1.96 (2)	2.795 (5)	166 (6)
O6—H6B···O2^{ii}	0.85 (2)	2.26 (4)	2.990 (5)	143 (5)
O7—H7A···O2^{ii}	0.86 (2)	1.85 (3)	2.649 (5)	154 (6)
O7—H7B···O3^{iii}	0.85 (2)	1.95 (2)	2.783 (5)	167 (6)
O8—H8A···N10	0.85 (2)	2.03 (3)	2.877 (6)	170 (8)
O8—H8B···N6^{iii}	0.86 (2)	2.08 (3)	2.909 (6)	163 (8)
O9—H9A···O8^{iv}	0.86 (2)	2.10 (2)	2.961 (6)	173 (8)
O9—H9B···O8^{v}	0.86 (2)	2.15 (3)	2.977 (7)	161 (8)

Symmetry codes: (i) x+1, y+1, z; (ii) x, y+1, z; (iii) x−1, y, z; (iv) −x+1, −y, −z+1; (v) x+1, y, z.

TABLE S5. Fitting parameters of Eq. **3** to experimental Δ_V (visible spectral range) and Δ_N (NIR spectral range) values.

Fitting Parameter	Δ_V	Δ_N
Δ_{1i}	2.2±0.1	9.1±0.1
Δ_{2i}	1.0±0.2	4.3±0.1
T_{0i} (K)	258±9	257±3
σ_i (K)	33±7	29±3
r^2	0.964	0.996

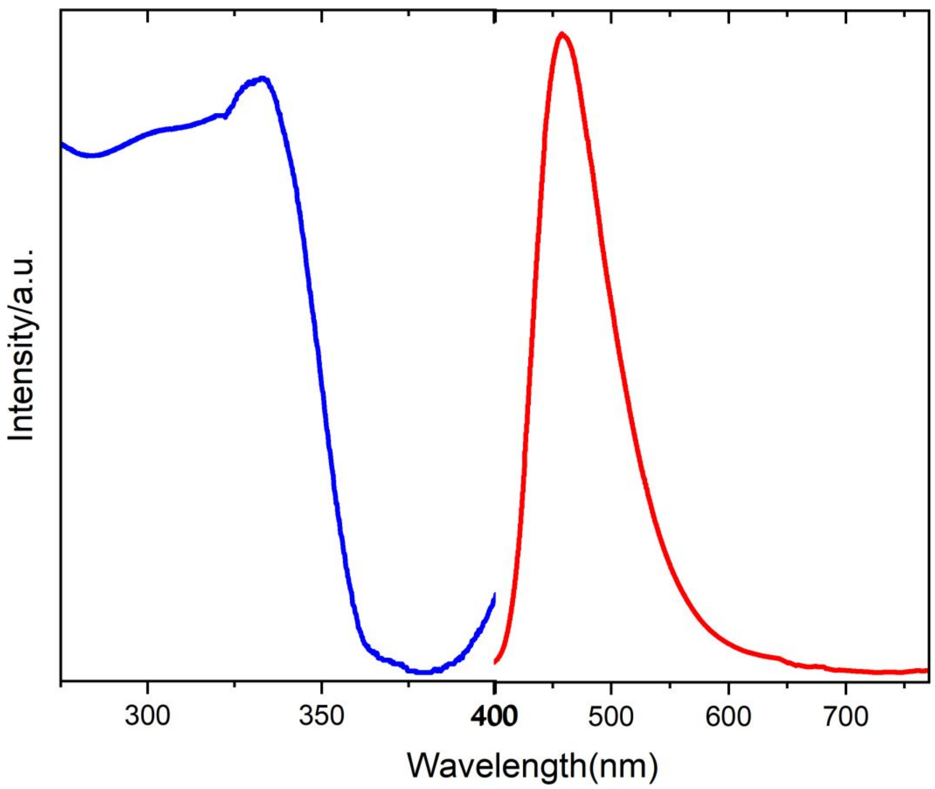

Figure **S3.** Emission (λ_{exc} = 330 nm, red line) and excitation (λ_{em}=457 nm, blue line) spectra of the ligand 4,4´-bpyO_2
